# Diffusion tensor imaging: Influence of segmentation on fiber tracking in the supraspinatus muscle–An inter-operator reliability analysis

**DOI:** 10.1371/journal.pone.0286280

**Published:** 2023-09-21

**Authors:** Sebastian Vetter, Hans-Peter Köhler, Pierre Hepp, Hanno Steinke, Stefan Schleifenbaum, Jan Theopold, Simon Kiem, Maren Witt, Jeanette Henkelmann, Christian Roth

**Affiliations:** 1 Sports Faculty Department of Biomechanics in Sports, Leipzig University, Leipzig, Germany; 2 Department of Orthopedics, Trauma and Plastic Surgery, Universitätsklinikum, Leipzig University, Leipzig, Germany; 3 Department of Anatomy, Universitätsklinikum, Leipzig University, Leipzig, Germany; 4 Institute of Sport and Motion Science, University of Stuttgart, Stuttgart, Germany; 5 Clinics of Diagnostic and Interventional Radiology, Leipzig University, Leipzig, Germany; National Institute of Biomedical Innovation Health and Nutrition, JAPAN

## Abstract

The ability of muscle to generate force depends on its architecture and health condition. MR-based diffusion tensor imaging of muscle (mDTI) is an innovative approach for showing the fiber arrangement for the whole muscle volume. For accurate calculations of fiber metrics, muscle segmentation prior to tractography is regarded as necessary. Since segmentation is known to be operator dependent, it is important to understand how segmentation affects tractography. The aim of this study was to compare the results of deterministic fiber tracking based on muscle models generated by two independent operators. In addition, this study compares the results with a segmentation-free approach. Fifteen subjects underwent mDTI of the right shoulder. The results showed that mDTI can be successfully applied to complex joints such as the human shoulder. Furthermore, operator segmentation did not influence the results of fiber tracking and fascicle length (FL), fiber volume (FV), fractional anisotropy (FA), axial diffusivity (AD), radial diffusivity (RD), and mean diffusivity (MD) showed excellent intraclass correlation estimates (≥ 0.975). As an exploratory approach, the segmentation-free fiber tracking showed significant differences in terms of mean fascicle length. Based on these findings, we conclude that tractography is not sensitive to small deviations in muscle segmentation. Furthermore, it implies that mDTI and automatic segmentation approaches or even a segmentation-free analysis can be considered for evaluation of muscle architecture.

## Introduction

Muscle architecture is a primary determinant of its function. A dramatic change in muscle fiber arrangement occurs with changes in sarcomeres [1–3]. The addition of sarcomeres in series lengthens the muscle fascicle [4], increases the shortening velocity [5], develops the range of motion [6] and supports force buffering and energy absorption during decelerative motion [7]. In practice, a longer FL is positively correlated with sport-specific performance [8–11]. Consequently, knee flexor weakness and a shorter FL have been shown to increase the risk of injury [12–15]. Therefore, FL is a primary parameter to determine the muscle architecture and the potential force output of the muscle. Since science and practice have paid much attention to the volume and cross-sectional area of a muscle, research into whole muscle fiber architecture has long been underrepresented and undervalued [16, 17].

To investigate muscle architecture, the most common non-invasive method is ultrasound imaging. Ultrasound appears to be a convenient, robust, and relatively inexpensive imaging technique [18]. However, ultrasound has several disadvantages: limited field of view, poor representation of all muscle fibers, difficult and time consuming to standardise [19]. This leads to difficulties in using ultrasound to access changes in muscle fiber arrangement over time [20, 21]. The fact that a change in muscle architecture is not uniform across all regions of the muscle [22], shows that an accurate method and a macroscopic view of a muscle’s fiber arrangement is important. Therefore, an innovative technology such as DTI may be of interest. DTI is a valid neuroscientific approach to measure the diffusion of water molecules along fibers to estimate potential cellular barriers representing architectural properties [23]. As muscles also contain a certain amount of water, an mDTI also provides a valid [24–26], reliable and robust [27, 28] calculation of muscle fiber metrics. In addition, this tool can be used to examine even more metrics that also describe the health status of the muscle [29–31]. Nevertheless, mDTI also has certain drawbacks: measurement of surrogate biomarkers, reconstruction of tracts varies and depends on algorithms and analysis methods, MR field strength, vendor and sequences [32] and is time-consuming to process data [18, 33].

In order to turn mDTI into a tool of practical significance, it is worth taking a look at the most common data processing steps. Among several processing methods commonly used in this field [34–36], the slice-by-slice segmentation of muscle seems to be the most laborious and operator-dependent step [37, 38]. Segmentation is necessary to extract a three-dimensional volume of interest (VOI) for each subject [38]. Since it is known that VOI-based tractography does not stop at the surface of the VOI [28] and that different segmentation techniques alter the tractography result for the lower limb muscles [37], the question is how different segmentation routines affect mDTI analysis for the human shoulder. Furthermore, it seems interesting to check the importance of segmentation for an accelerated mDTI approach. Therefore, this study aims to show the dependence of two different segmentation routines for segmentation-based analysis (SBA 1 and SBA 2) on the manually segmented muscle model volume (MV) and its impact on tractography. This allows the interpretation that multiple sides with different segmentation operators can produce similar results. Furthermore, in an exploratory way, we want to compare these results with a segmentation or model-free approach (MFA). For the reliability analysis, we focused on the calculation of muscle shape indices (MV, FL and FV) and conventional reported tensor metrics (FA, AD, RD and MD), which also reflect the arrangement and health status of a muscle [29, 30].

## Materials and methods

This study analysed a dataset from an interventional trial consisting of fifteen subjects who underwent MRI [39]. The protocol was approved by the local ethics committee (Leipzig University, No: 362/21-ek) and is registered in the German Register for Clinical Trials (DRKS00032375). The study was conducted in accordance with the relevant guidelines and regulations. Written informed consent was obtained from all subjects. The authors had no access to information that could identify individual participants during or after data collection.

### Sample

Fifteen healthy male subjects (24.1 ± 3.8 years of age; 183.7 ± 6.5 cm tall; 79.5 ± 7 kg body mass) were recruited for MRI in October 2021. Criteria for inclusion were: no history of muscle-nerve disease, no injuries, no discomfort or pain in the right shoulder, no resistance training five days prior to data collection, no regular medication.

### Data acquisition

Magnet resonance imaging (MRI) of the right shoulder was performed using a 3 Tesla MRI scanner (Siemens MAGNETOM Prisma Fit, Erlangen, Germany) with a dedicated 16-channel shoulder coil in the head-first supine position. The right shoulder was placed in a neutral position with the arm adducted and the hand supinated. The MR protocol consisted of a 3D coronal T1-weighted (T1w) and a sagittal DTI sequence from distal to proximal. The total scan time was approximately twelve minutes. The T1w sequence was acquired with the following parameters: repetition and echo time *T*_*R*_*/T*_*E*_ = 492/20 ms, slice thickness = 0.7 mm, flip angle = 120°, field of view FOV = 180 x 180 mm^2^, matrix = 256 x 256 mm^2^. For DTI a commercial Siemens 2D echo planar diffusion image sequence was acquired with the following parameters: repetition and echo time *T*_*R*_*/T*_*E*_ = 6100/69 ms, slice thickness = 4 mm, flip angle = 90°, field of view FOV = 240 x 240 mm^2^, matrix = 122 x 122 mm^2^, 48 diffusion sampling directions with *b* = 400 s/mm^2^. This diffusion sequence showed an in-plane resolution of 1.96721 x 1.96721 mm.

### Muscle segmentation

**Fig 1:**
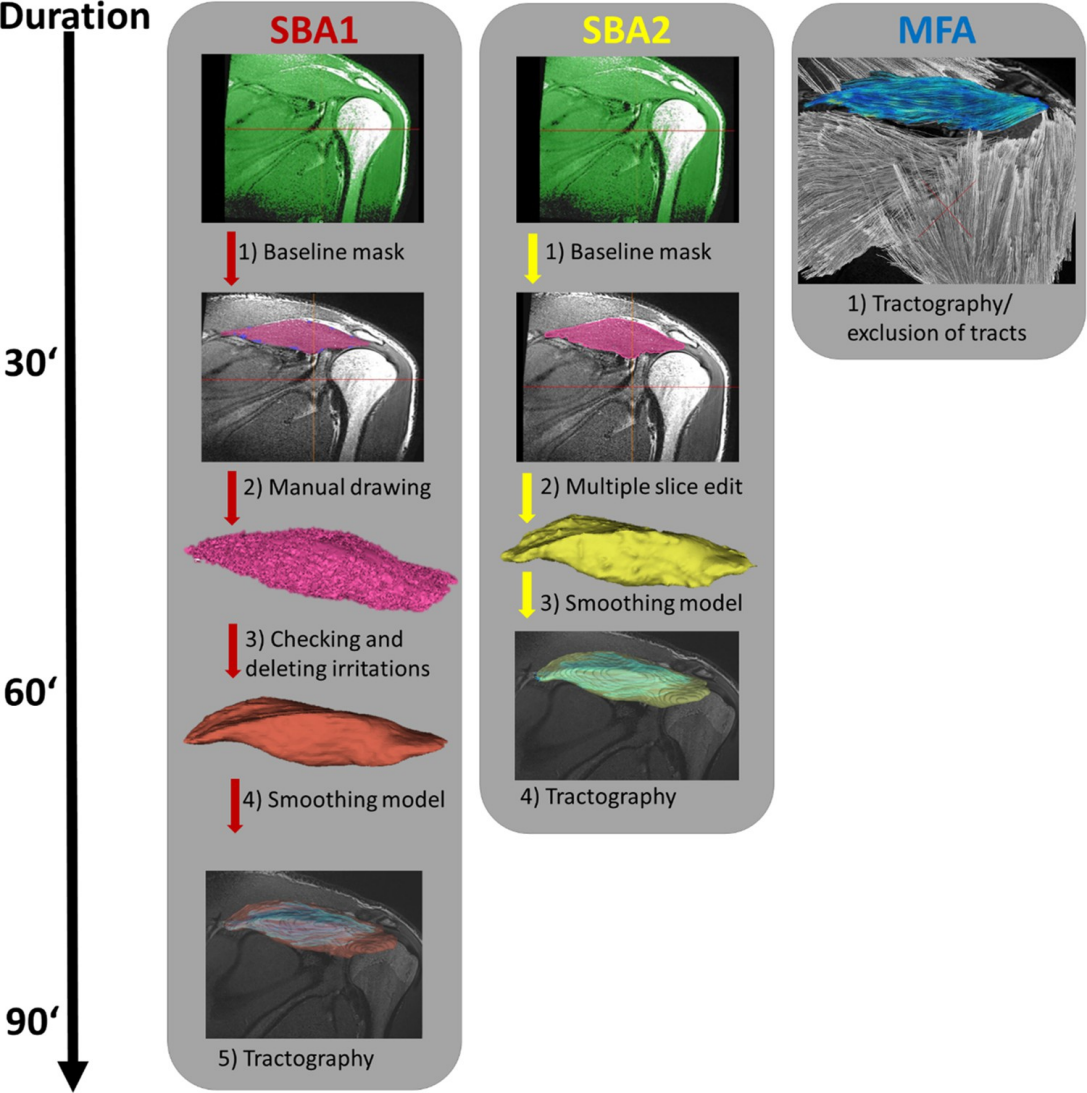
Workflow of methods. Workflow displays different processing steps and each methods duration in minutes (‘). Segmentation-based analysis by operator 1 (SBA 1) included four major segmentation steps, operator 2 (SBA 2) displayed three steps. Model-free analysis (MFA) did not include a segmentation and used the entire field of view as seeding area for deterministic fiber tracking. Within MFA the red cross symbolises the manual exclusion of tracts outside of the highlighted supraspinatus muscle (blue color).

Manual segmentation was based on common mDTI methods described previously. Segmentation was performed using Mimics Materialise (v.24.0, Leuven, Belgium). Two independent operators (SBA 1 and SBA 2) segmented each supraspinatus muscle. Segmentation was based on the recorded T1w sequence with 176 slices. To compare individual differences in segmentation, each operator generated an individual segmentation routine for the whole data set. The first segmentation step was to generate a base mask by setting a threshold on the grey values and brightness of the pixels to separate muscle-tendons from bony structures. Then, both operators split the basic muscle mask to separate the supraspinatus muscle from the other surrounding tissues and to proceed with manual segmentation and correction. While SBA 1 preferred manual segmentation, operator two (SBA 2) focused on the integrated interslice interpolation tool for multiple-slice edit (Fig 1). However, both operators used integrated Software tools such as thresholding, region growing, filling hole and interslice interpolation to standardize segmentation and reduce the total duration for segmentation. Finally, each VOI was smoothed with first-order Laplacian (10 iterations, smoothing factor 1.0) and exported as 3D triangular meshes STL file.

### DTI data processing and fiber tracking

DSI Studio (v. 3^th^ of December 2021. http://dsi-studio.labsolver.org) was used for DTI processing, deterministic fiber tracking and tract calculations. To perform tractography for the supraspinatus muscle, we registered and resampled the DTI images to the T1w images. The quality of the DTI and FA maps was first visually checked by two experts using DSI Studio. In addition, the DTI images were corrected for motion and eddy current distortion using DSI Studio’s integrated FSL eddy current correction. To ensure plausible fiber tracking results, we used the following stopping criteria recommended: maximum angle between tract segments 15°, 20 mm ≤ tract length ≤ 130 mm; step size = 1.5 m These settings were oriented to FL results of cadaveric dissections [40] and recommendations for deterministic muscle fiber tracking stopping criteria [41–43].

Fiber tracking was then performed either within a model VOI (SBA methods) or for the entire DTI images without using a segmented model (MFA). Tensor metrics were calculated based on DTI as a model-based tracking method using DWI with a b-value less than 1750s/mm^3^ and a 4^th^-order Runge-Kutta tracking algorithm [44]. After a reconstruction of ~10.000 tracts for the supraspinatus muscle, tractography was terminated and duplicates were deleted. VOI was negated and set as terminative region for tractography [45]. Because MFA used the entire DWI field of view as the seeding area for tractography, we removed all tracts that ran outside the supraspinatus muscle based on the t1w images used as background for anatomical orientation. In SBA and MFA, interfering reconstructed tracts were manually removed if they showed an orientation perpendicular to the line of action of the muscle.

Finally, DTI tensor parameters (FA, AD, MD and RD) and muscle parameters (MV, FL and FV) were calculated based on a deterministic fiber tracking algorithm [46] and specific tracking strategies [31] using DSI Studio. FV was calculated as the absolute volume of the voxels used for tensor calculation and fiber reconstruction. Since MFA did not include a muscle segmentation step, it took approximately 30 minutes. In contrast, SBA 1 and SBA 2, including segmentation, took approximately 90 and 60 minutes respectively.

### Data analysis

SPSS v.27 (IB Armonk. New York. USA) was used for statistics. Figures were generated using MATLAB v.R2022a (MathWorks. Natick. USA). Descriptive results were based on the calculation of mean values and the standard deviation (±). As the human shoulder has complex structures and very different muscle shapes, two raters visually inspected the image slices for quality. If the inspection or statistics revealed outliers, they were excluded from further analysis. Outliers were defined visually based on distribution and box plots and calculation of z-transformed values. If they exceeded 3.0 within a metric, they were excluded. After checking the quality of the data, repeated measures analysis of variance (rmANOVA) was used to show overall differences in means between methods. Greenhouse-Geisser correction was applied if appropriate. When a main effect was found, Bonferroni-corrected post-hoc comparisons were performed. In addition, interrater-reliability between all methods (SBA 1, SBA 2 and MFA) was analysed using an intraclass correlation coefficient (ICC) for each mDTI (FA, MD, AD, RD) and muscle (FL, FV, and MV) parameter. ICC estimates and their 95%-confidence intervals (CI) were based on a single-rating, absolute-agreement and a two-way random effects-model (2.2). ICC values less than 0.5 were considered as poor, 0.5 to 0.75 as moderate, 0.75 to 0.9 as good, and greater than 0.9 as excellent [47]. The significance level was set at *p* < .05. Bland-Altman plots were calculated to show the limits of agreement.

## Results

All MR scans could be used for data processing and were included in the analysis. Segmentation for SBA 1 and SBA 2 was performed by two independent operators. SBA 1, SBA 2 and MFA were applied to the human supraspinatus muscle of the human right shoulder.

**Fig 2:**
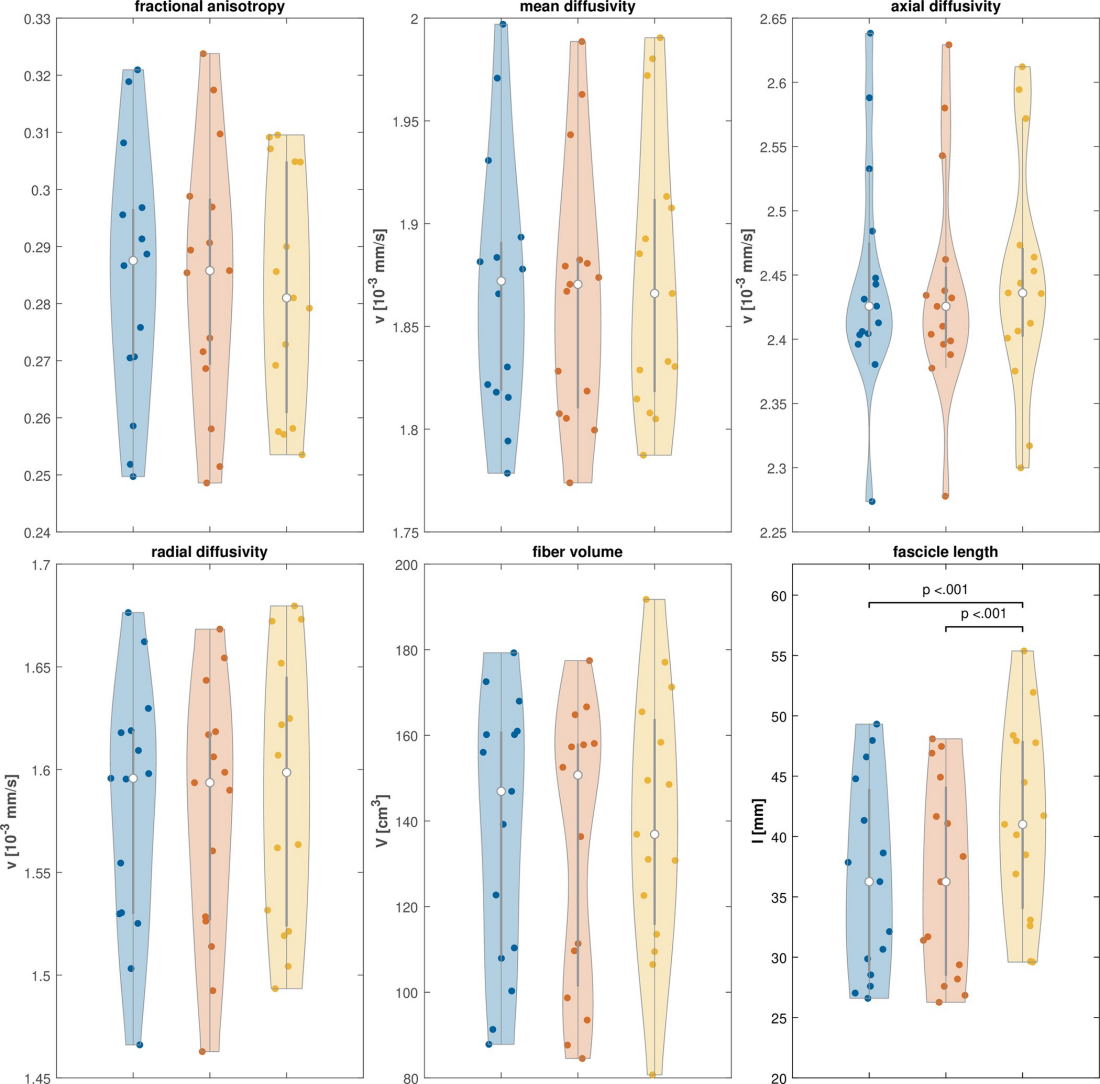
Violin plots and mean differences between methods. Violin plots show mean differences between segmentation-based analysis (SBA 1 [blue] and SBA 2 [red]) and model-free analysis (MFA, yellow).

The descriptive results (Table 1) show similar values with low variance for mDTI indices (CV ≤ 8.02). In contrast, FL and FV show higher variance (CV ≥ 19.45) and mean differences between subjects and methods (FL mean = 36.34–41.27 mm). Greenhouse Geyser corrected rmANOVA were used to assess differences and showed a main model effect only for FL (F_1.135, 15.890_ = 22.645; *p* < 0.001; η^2^_p_ = 0.618). Further post-hoc paired *t*-tests (Fig 2) revealed significant differences in segmentations with respect to MV (t_14_ = 6.407; *p* = 0.001), but no significant differences in FL between the two SBA methods (Fig 2).

**Table 1:** Descriptive results.

Metric	SBA 1					SBA 2					MFA				
	Mean	±	Min.	Max.	CV %	Mean	±	Min.	Max.	CV %	Mean	±	Min.	Max.	CV %
FA	0.28	0.02	0.25	0.32	7.76	0.28	0.02	0.25	0.32	8.02	0.28	0.02	0.25	0.31	7.38
MD	1.87	0.06	1.78	2.00	3.34	1.87	0.06	1.77	1.99	3.35	1.87	0.07	1.79	1.99	3.59
AD	2.44	0.09	2.27	2.64	3.62	2.44	0.09	2.28	2.63	3.56	2.45	0.09	2.30	2.61	3.70
RD	1.58	0.06	1.47	1.68	3.81	1.58	0.06	1.46	1.67	3.90	1.59	0.07	1.49	1.68	4.10
FL	36.34	8.12	26.59	49.31	22.35	36.41	8.16	26.27	48.09	22.40	41.27	8.03	29.60	55.37	19.45
FV	137.6	31.3	87.8	179.3	22.8	133.8	32.5	84.5	177.5	24.3	139.6	30.4	80.7	191.7	21.8

Columns show the mean value, standard deviation (±), minimum and maximum values and coefficient of variation (CV %) for each method, segmentation-based analysis (SBA) and model-free analysis (MFA). The rows show fractional anisotropy (FA,), axial diffusivity (AD), radial diffusivity (RD), and mean diffusivity (MD) in 10^−3^ mm/s, fascicle length (FL) in mm, and fiber volume (FV) in mm^3^.

When assessing operator dependence for fiber tracking outcome measures (Table 2) the ICC was found to be good (≥ 0.751). Agreement was highest between SBA 1 and SBA 2, ranging from ICC estimates between 0.975 to 0.997. As an explorative approach, the comparison of SBA to MFA revealed an ICC of 0.751 for FL. Ignoring the results for FL, the ICC values for MFA varied between 0.840 (FV) and 0.930 (MD).

**Table 2:** Interrater reliability.

Parameter	Methods	ICC	95% CI	
FA	Overall	0.935	0.856	0.976
	SBA 1 vs SBA 2	0.997	0.992	0.999
	SBA 1 vs. MFA	0.901	0.736	0.965
	SBA 2 vs. MFA	0.903	0.741	0.966
MD	Overall	0.950	0.887	0.981
	SBA 1 vs SBA 2	0.994	0.979	0.998
	SBA 1 vs. MFA	0.928	0.804	0.975
	SBA 2 vs. MFA	0.930	0.808	0.976
AD	Overall	0.948	0.883	0.981
	SBA 1 vs SBA 2	0.995	0.980	0.999
	SBA 1 vs. MFA	0.923	0.787	0.974
	SBA 2 vs. MFA	0.927	0.801	0.975
RD	Overall	0.944	0.875	0.979
	SBA 1 vs SBA 2	0.994	0.981	0.998
	SBA 1 vs. MFA	0.920	0.786	0.972
	SBA 2 vs. MFA	0.923	0.783	0.973
FL	Overall	0.823	0.401	0.944
	SBA 1 vs SBA 2	0.990	0.972	0.997
	SBA 1 vs. MFA	0.751	0.012	0.933
	SBA 2 vs. MFA	0.752	0.000	0.932
FV	Overall	0.888	0.761	0.957
	SBA 1 vs SBA 2	0.975	0.908	0.992
	SBA 1 vs. MFA	0.840	0.588	0.943
	SBA 2 vs. MFA	0.843	0.604	0.944

Intraclass coefficient (ICC) and its confidence intervals (CI) for segmentation-based analysis (SBA 1 and SBA 2) and model-free analysis (MFA) for the parameters fractional anisotropy (FA,), axial diffusivity (AD), radial diffusivity (RD), and mean diffusivity (MD) in 10^−3^ mm/s, fascicle length (FL) in mm, and fiber volume (FV) in mm^3^.

**Fig 3:**
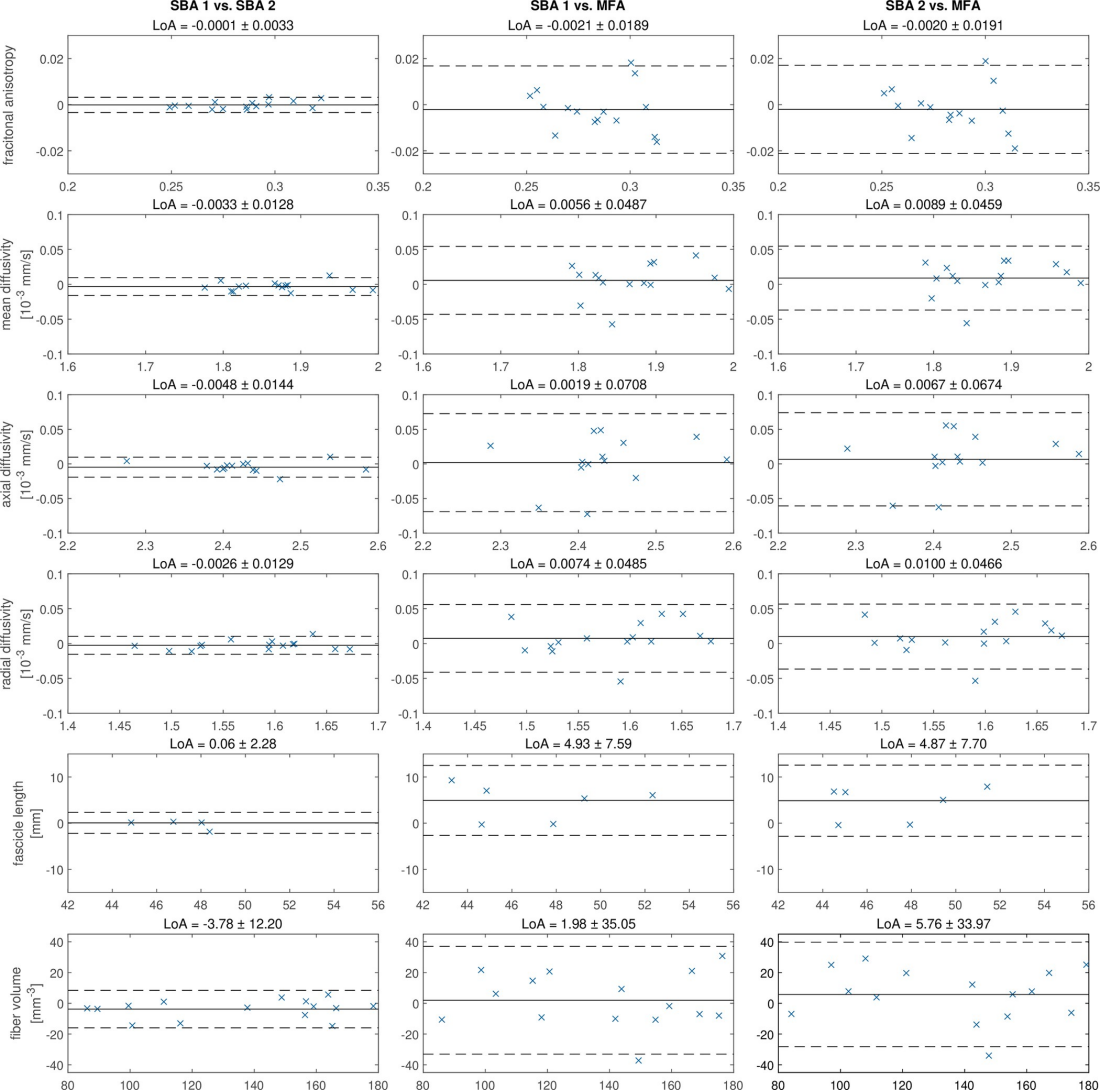
Bland-Altman plots and limits of agreement. Bland-Altman plots for interrater-reliability for segmentation-based analysis by two different operators (SBA 1 and SBA 2). and model-free analysis (MFA). The x-coordinate shows the mean of two methods and the y-coordinate shows the value differences between each method. The middle black line shows the mean of the paired differences. The dashed line presents the limits of agreement.

Bland-Altman plots (Fig 3) display the mean and the difference of each method within the limits of agreement. The lowest limits of agreement were found for SBA 1 and SBA 2, with the exception of FV(3.78 ± 12.20). The highest limits of agreement show comparisons between SBA and MFA similar for FL (4.87 and 4.93 for SBA 2 vs MFA) but different for the metric FV (1.98 and 5.76 for SBA 2 vs MFA).

## Discussion

The main aim of this study was to investigate how two different and independent operators, who developed their own segmentation routine, affect the tractography. The results show that two different operators in segmentation resulted in different MV with no influence on fiber tracking and with an excellent ICC (≥ 0.975) [47]. This is consistent with Forsting et al. [37] investigating differences between independent raters using pre-specified segmentation routines for the lower limb muscles. In an explorative comparison of SBA methods to a more convenient model-free fiber tracking (MFA) approach, muscle parameters displayed a good ICC (0.751–0.843) and excellent ICC for the mDTI indices (0.901 and 0.93) [47]. Bland-Altman plots (Fig 3) showed no major anomalies in the data and no over- or under-estimation of parameters between SBA and MFA.

Due to the anatomy of the supraspinatus muscle and its tendonous structure, differences in MV are to be expected. As the task of both operators was to segment only the muscle and to develop their own segmentation routine independently, they were first introduced to segmentation. The aim was to check if the two operators approached segmentation differently and if this influences a fiber tracking result since it allows the conclusion that multiple sides can reveal similar results without interaction between raters. As expected, the two operators’ routines differed in the number of processing steps, duration (30–45 minutes), details of manual drawing, and how they defined the muscle-tendon interface (Fig 1). In terms of daily clinical routine, this case may reflect reality, as there is no standardised segmentation routine trained and checked by only one expert [38]. Based on this assumption, ICC estimates were calculated in this study using a two-way random effects model [37, 38]. Therefore, the findings reported might be generalised to any random operator performing a muscle segmentation regardless of the operator’s experience and the level of detail in manual segmentation.

The observed differences in FL between SBA and MFA could be attributed to several reasons: Based on the findings of Bolsterlee et al. [26, 34] and Damon et al. [24, 48] explaining that the most valid tracts are found at the belly of the muscle, we removed tracts that obviously crossed the muscle boundaries in MFA. This may have removed relevant fibers in MFA that were included in SBA. Secondly, MFA used the entire DTI image as the seeding area for fiber tracking, which may result in different density and distribution of tracts [37]. However, this makes it difficult to compare muscle metrics from SBA to MFA and it may be even more interesting to see how accurately these approaches detect changes in muscle architecture over time. Furthermore, cadaveric dissections show that the mean FL for the human supraspinatus muscle can vary between 28 mm and 83 mm [40] depending on the region of interest and the number of fascicles measured [17]. This indicates that FL should be compared carefully and in respect to the recruited subjects and the applied analysis methods.

Since the physiological cross-sectional area (PCSA) is commonly used to calculate the force-generating potential of a muscle [16, 49], FV may also be of interest to calculate the force-generating potential of a muscle, since it represents the distribution of fibers within the muscle. In this study, FV showed high variance (CV ≥ 21.8) due to very different MV between subjects (68–95 mm^3^ in SBA 2). However, the FV was not significantly different between methods (*p* > 0.05) and showed a good ICC (≥ 0.840). Results for FA, MD, AD and RD are comparable to Forsting et al. [37]. Furthermore, our FA and RD values revealed that the recruited subjects did not have irregular values as shown for injured subjects [29, 30]. This is important to demonstrate as it appears that muscle tears have a significant effect on FL [12–15] and DTI indices [29, 30].

DTI is an innovative opportunity to have a detailed look at a muscle’s fiber architecture. Nevertheless, it has some inherent difficulties. To generate DTI, the diffusion coefficient of water is measured and a mathematical representation of its diffusion is generated in three dimensions. Diffusion should be measured in at least six directions to represent the diffusion anisotropy of the muscle. Fiber tracking therefore maps the connectivity between different points within a biological VOI [48]. However, mDTI acquisition varies between studies in terms of sequences and its settings [48], segmentations [37, 38] and data analysis methods [35, 36]. This leads to incomparable results between studies [32]. Furthermore, since diffusion data are acquired in a macroscopic range to measure the water diffusion and to estimate fibers, a fiber length is only an estimation of the actual fiber length [50]. Therefore, a careful plausibility check based on a sum of muscle and DTI metrics is required to allow interpretation of the reconstructed tracts as muscle fibers. While other non-invasive technologies such as conventional 2D ultrasound or micro-computed tomography do not require fiber reconstruction, they appear to be more convenient for daily clinical routine in terms of injury and disease. However, it should be noted that such approaches usually measure a small number of fascicles even in resistance training studies [51], which has never been quantitatively tested to represent the muscle architecture accurately [16]. As shown by Charles et al. [17], this may lead to large errors in the interpretation of the whole muscle architecture. Therefore, mDTI appears to be more valid when considering the three-dimensional architecture of the whole muscle. In addition, mDTI can be interpreted as an accurate method for assessing changes after interventions [52].

Comparing mDTI with ultrasound also raises the issue of test economics and feasibility in research and clinical contexts. As mentioned, mDTI involves very complex data processing, which guided the subject of this study. To be precise, usually mDTI contains the following processing steps recommended [34–36]: (1) manual segmentation of target muscle based on anatomical sequences; (2a) resampling and registration of DTI images to T1w images; (2b) quality control of DTI; (2c) definition of VOI-specific stopping criteria for fiber tracking; (2d) integration of the segmented muscle model as VOI; (2e) fiber tracking; (3a) generating a surface model (3b) importing and translating the coordinates of identified tracts onto the surface model; (3c) calculation of muscle parameters. This is a typical workflow in which mDTI appears to be less competitive than ultrasound imaging. Nevertheless, we know that mDTI and ultrasound should be compared carefully [53]. As ultrasound is widely used as a practical imaging tool to detect major irregularities within anatomical structures, mDTI seems to be more important for research tasks due to a high amount of data that can be collected [35]. However, if ultrasound imaging has a higher degree of standardisation in terms of positioning and analysis [19], it may result in a similar level of effort to mDTI. Since a few studies have shown that changes in mDTI parameters can help to detect muscle tears [29, 30], we assume that mDTI may be of clinical relevance. The fact that muscle architecture measurements with mDTI have been shown to be reliable [27], and robust [28] gives mDTI advantages over ultrasound imaging [21]. The fact that SBA 1 and SBA 2 did differ in terms of effort and MV but not in terms of fiber tracking outcome, suggests that automatic segmentation approaches [54, 55] may be sufficient for accurate calculations of FL.

### Limitations

This study used original Siemens DTI sequences, which have slightly different settings to those recommended for mDTI [41]. This increased the risk of inappropriate mDTI data and incomparable results. In addition, the quality of the data also depends on the size and complexity of the target structure [41]. As the individual anatomy and complexity of the human shoulder complicates DTI acquisition, we observed very heterogeneous data quality, resulting in a large variance between datasets. Furthermore, the analysis of supraspinatus muscle also revealed difficulties. As cadaveric dissections have shown, the supraspinatus muscle has a very different fiber orientation within the anterior or posterior superficial, middle and deep regions [40]. Since it is known that a tractography can reveal disoriented tracts and tracts that cross the muscle boundaries [28], we were not able to detect in detail the different fiber orientation within the target muscle. With respect to DTI settings, the chosen step size as a stopping criteria for fiber tracking was recommended by Forsting et al. [42]. However, a smaller step size compared to the voxel size leads to interpolations, which requires even more caution in interpreting the results. Furthermore, as our commercial sequences showed untouched settings to test feasibility in a clinical context, this led to difficulties in resampling and registering the DTI images to the T1w images used for segmentation. We therefore decided not to calculate other muscle parameters such as pennation angle, which has also been discussed elsewhere [35].

Another point to discuss is the estimation of fascicle length in this study. First, VOI was extracted using Mimics Materialise for muscle segmentation, which has different segmentation characteristics and may cause difficulties when compared to other mDTI studies using other segmentation tools [34–36]. Second, as explained in the Methods section, tractography in SBA was performed based on a negated VOI. Since this negated VOI was set as the end region for fiber tracing, tracts running outside the target muscle should not be reconstructed as tracts. This method is based on Bolsterlee and colleagues [45] who, similar to other mDTI studies [34–36], then determined fascicle length by calculating the Euclidean distance between the endpoints of the tracts using MATLAB. In contrast, this study estimated fascicle length using the built-in statistics tool in DSI Studio [46]. However, this method showed more opportunities for standardization and objectivity and less data transfer to different software, thus supporting clinical relevance.

In conclusion, all of these methodological considerations and the fact that the interrater analysis is based on two operators segmenting muscles in a small sample of highly active male student athletes indicate that the results should be interpreted with caution alongside other mDTI studies [34–36].

## Conclusion

This study shows that different segmentation routines do not influence tractography for the human shoulder supraspinatus muscle. This is in concordance to a study for the lower limb [37]. Furthermore, tractography without a segmentation step (MFA) shows good ICC estimates. Therefore, SBA or MFA may be advantageous depending on the needs. In the case of a muscle lesion [29, 30], MFA can be used as a first and quick decision basis for further treatment. On the other hand, SBA can be used to show training-induced changes and for a more precise analysis of muscle parameters. In terms of test economy, MFA may compete with ultrasound imaging methods. While SBA 1 and SBA 2 differed in time and detail, but not in tractography results, automatic segmentation approaches can be considered to maintain the accuracy of FL calculations and speed up analysis. As the human shoulder joint is known to be a challenge for any imaging technique, the results of mDTI were very promising and detailed. Therefore, mDTI can claim practical relevance. The evaluated mDTI processing methods could form the basis for further interventional studies and future methods. MFA is an acceptable and reliable mDTI analysis method that shortens segmentation to significantly reduce the post-processing pipeline to assess the muscle fiber architecture and muscle health as quickly as currently possible. Therefore, mDTI seems worth considering as a standard sequence within a musculoskeletal MR application in a clinical context. However, future studies need to clarify whether further post-processing steps can be shortened or automated for daily clinical use. Furthermore, it remains unclear whether MFA could be applied to other muscles with different fiber orientations. To our knowledge, this is the first study using mDTI in the human shoulder in this context.
